# Nature versus nurture—on the origins of a specious argument

**DOI:** 10.1093/exposome/osac005

**Published:** 2022-08-02

**Authors:** Robert O Wright

**Affiliations:** Department of Environmental Medicine and Public Health, Icahn School of Medicine at Mount Sinai, New York, NY, USA

**Keywords:** exposomics, complex diseases, genomics, gene–environment interactions, missing heritability

## Abstract

The concept of heritability parses out genetic and environmental causes of diseases and does not fit the underlying biology of complex diseases that arise from interactions among genetics and environment. Exposomics places environment on a similar scale as genomics and allows for more modern research approaches that estimate time-varying genome by exposome interactions. By addressing the biological underpinnings of disease comprehensively, we will find the “missing heritability” which is not solely based on genetic variation but is instead driven by time, life stage, and geographic variability in our exposome as it interacts with our genome.

Heritability studies are among the more common projects cited in the lay press. In most cases, the article will say something similar to “New Research shows that Disease ‘X’ is Genetic.” The lay press’s focus on heritability has contributed to a broad genocentric view of initiatives such as precision medicine.^1^^,^^2^ Heritability studies are typically performed in twins, both identical and fraternal. Because identical twins have the exact same genetic variation, and fraternal twins share an average of 50% of their genetic variation, the rates of a disease in both types of twins can be compared with the primary goal of estimating the genetic contribution. In theory, the goal could be to estimate the environmental contribution, but if that has been emphasized, it is the exception, not the rule. Of course, a lot of assumptions have to be made about how to divide causation between genetics and environment, since more than just genes are shared between twins. All twins share an identical environment in pregnancy and even after birth they share very similar environments. The division of what arises from shared environment versus shared genetics is subjective at best. Regardless, these studies divide disease causation to a percentage due to genetics and a percentage due to environment. Although there have been many calls to include environment as a critical, modifiable contributor to preventing disease, the ‘elephant in the room’ of personalized medicine is that the field has grown ever more genocentric over time.^2^ With the advent of exposomics, the field is beginning to shift toward an understanding that environment matters critically in all diseases as well.

Because they are simple to understand and explain, heritability studies make good soundbites. For this reason, we should consider the impact they have on the general public. If a newspaper article states that a study showed that asthma is 82% genetic^3^—most people would think this meant that out of every 100 people with asthma, 82 of them got it because of their genes and 18 got it because of environment. *But it doesn’t mean that.* In fact, there is no known “gene” or “cluster of genes” that *causes* asthma. Genetic variants provide increased risk, not cause. We also know that environment (pollen, animal dander, infections, air pollution, stress, and many, many others) plays a role as they all can trigger asthma attacks and most have been associated with its onset. This heritability study could make the public think that environment is not as important to asthma as genetics. Yet all asthma interventions, drugs, inhalers, allergen mitigation, etc. are designed to address environment. Gene therapy is not around the corner for asthma, nor is it likely to ever be.

Simplicity can be good for communicating scientific information, but oversimplification can be damaging. Biology is far too complicated to be boiled down to two percentages attributing cause to genes versus environment. Even more importantly, the real problem with the concept of heritability is that it assumes that our genes and our environment don’t work together. That’s the major flawed assumption—that they can be divided as causes. Focusing on heritability prevents us from moving science forward to understand what is really happening. Furthermore, we not only assume they are independent, we also routinely use language that suggests they work against each other (ie, Nature versus Nurture), and by doing so we create false impressions. *Genes and environment always work hand in hand*. How can anyone ever decide how much of a percentage to give out to each? It’s like deciding what percent of the Beatles success we should give to Ringo.

So how did we get here? The origin of this problem comes from that simple phrase we all learned in high school—‘nature versus nurture’. We use it to convey the idea that genetics plays a role in our health and so does environment. In theory that seems like a good communication strategy, except our language conveys a different message. The “versus” in Nature versus Nurture implies a contest—like a prize fight. Nature versus Nurture was first coined in the mid-1800s by an English statistician—Francis Galton (interestingly, he was Charles Darwin’s cousin) while writing about the influence of genetics and environment on intelligence. His 19th century concept is badly outdated. We know environment is key to intelligence and to test taking ability. In fact, the Flynn effect refers to the increase in population intelligence quotient (IQ) scores observed throughout the 20th century of about three IQ points per decade. Rapid changes like that cannot be explained by genetics.^4^ “Nature versus nurture” has been kept alive far too long and continues to make us pick a side—genetics or environment.

In fact, Galton was positing that genes create or select an environment of wealth and privilege which is counter to Darwin’s theory of Natural Selection. While most people think of natural selection as a genetic theory, it is actually a theory about how genes and environment interact. Under natural selection, genes are selected if they provide an advantage for reproduction in a given environment. If we don’t reproduce, our genes “die” so to speak, as they cease to exist in subsequent generations. If our environment were ever to change to be harsh enough to kill a large number of us at an early age, perhaps due to famine, some genes might provide survival advantages. Maybe your genes would allow for a higher absorption of food during a famine, so you might survive longer than others, but famine hasn’t happened in America or Britain for a very long time and those genetic traits have no added value outside of famine. Galton was an aristocrat who believed that his genes selected his environment of wealth and education and that his progeny’s genes would do the same. Natural selection *actually posits the opposite*. Environment selects for genes that better survive in harsh conditions. Under natural selection, being poor and oppressed should select more adaptive, higher functioning traits over time than genes passed down multiple generations in a family that is rich through inherited wealth. In fact, many environmental factors, such as the great wealth of Galton’s family, are also inherited. The impact of his inherited wealth cannot be easily disentangled from the genetic variants that by chance came with it. His wealth made it seem to him like his genes functioned more highly than those in people without wealth, but his genes were just there for the ride, and were no different or better than working class people. Inherited wealth will make anyone’s inherited genes look better than they actually are.

We can even illustrate how environment and genes always interact, by using genetic diseases. Phenylketonuria or PKU—for short. It is a genetic disorder that arises from a mutation in the phenylalanine hydroxylase gene. If a person has two copies of the mutated gene they cannot properly metabolize phenylalanine, an amino acid, after it is ingested. Toxic forms of metabolism accumulate and can damage the developing brain. Untreated, PKU can lead to intellectual disability, seizures, and behavioral problems. However, the disease won’t occur if a newborn baby is given an *environmental* intervention—a special low phenylalanine formula. The same is true for any genetic disease, all genes have something in the environment that acts as a substrate. Sometimes that substrate is so common or essential that it cannot be easily regulated or avoided and penetrance is high (cystic fibrosis and electrolytes) or sometimes it is so variable in different environments that penetrance is highly variable (hemochromatosis and iron). More recent publications have stressed that heritability is not deterministic and *is expected* to vary across populations as the prevalence of genetic variants and environment factors nearly always vary across different populations and the genetic and environmental variance is tied to their prevalence.^5^

What would happen if we studied the heritability of PKU using twins. While some may get missed during screening, the majority of fraternal twins with the PKU genotype would be treated, many of the identical twins would be too. This means the heritability of a disease we label as 100% genetic would be <100% if we did a twin study of PKU and developmental delays. That alone tells us that heritability is not dependent only on genetics. Ken Rothman, a famous epidemiologist, once wrote “all diseases are 100% genetic and 100% environmental.”^6^ In other words, they are not fighting, they are working together and always do. While PKU has a relatively uncomplicated biological cause, complex diseases have many genetic and environmental risk factors, with the environmental factors being potentially time varying. Attempts at estimating shared environment in early life have been made in Twin Studies, which is a welcomed development.^7^ However, we have not yet invested as a society in developing the technologies needed to measure the exposome on a scale similar to the genome, although many of the geospatial, instrumentation, and analytical infrastructure exists. Until we commit the resources to measure the exposome, we will have continue to fall back on dichotomous estimates of genetic and environmental contributors to disease. We need to measure the “E” in the ubiquitous “G by E” interactions that drive health and disease, not measuring G by E interactions is the reason genetics has not made our society healthier. We need to understand the environmental factors our genes interact with, otherwise nothing will ever change.

So the next time a study breaks down a disease into percentages of genetic and environmental causes, we should ask—can genes ever operate by themselves? Or do they operate by interacting with the world in which we live? There is no example of even one gene that doesn’t ultimately operate by interacting with something that we ingested, inhaled, or acquired then synthesized in some way inside our body. Every gene needs a substrate for the protein it encodes. That substrate comes from our environment—genes need environment to operate. If we expand our measures of the environment to the exposome, just as genetics expanded to genomics, we can begin understand the complex time-varying ways the genome and exposome interact.

Maybe this concept was best expressed by the neuropsychologist Donald Hebb, who researched language acquisition and learning in children. When he was asked ‘Which contributes more to personality—nature or nurture?’ he answered ‘Which contributes more to the area of a rectangle, its length or its width^8^?’

## Funding

This work was supported in part by the NIH grants P30ES023515, U2CES026561, and U2CES030859.

**Conflict of interest statement**: None declared.
